# Genomic prediction of survival time in a population of brown laying hens showing cannibalistic behavior

**DOI:** 10.1186/s12711-016-0247-4

**Published:** 2016-09-13

**Authors:** Setegn W. Alemu, Mario P. L. Calus, William M. Muir, Katrijn Peeters, Addie Vereijken, Piter Bijma

**Affiliations:** 1 Department of Molecular Biology and Genetics, Center for Quantitative Genetics and Genomics, Aarhus University, 8830 Tjele, Denmark; 2 Animal Breeding and Genomics Centre, Wageningen University and Research, 6700 AH Wageningen, The Netherlands; 3 Research and Technology Centre, Hendrix Genetics, 5831 CK Boxmeer, The Netherlands; 4 Department of Animal Science, Purdue University, West Lafayette, IN 47907-1151 USA

## Abstract

**Background:**

Mortality due to cannibalism causes both economic and welfare problems in laying hens. To limit mortality due to cannibalism, laying hens are often beak-trimmed, which is undesirable for animal welfare reasons. Genetic selection is an alternative strategy to increase survival and is more efficient by taking heritable variation that originates from social interactions into account, which are modelled as the so-called indirect genetic effects (IGE). Despite the considerable heritable variation in survival time due to IGE, genetic improvement of survival time in laying hens is still challenging because the detected heritable variation of the trait with IGE is still limited, ranging from 0.06 to 0.26, and individuals that are still alive at the end of the recording period are censored. Furthermore, survival time records are available late in life and only on females. To cope with these challenges, we tested the hypothesis that genomic prediction increases the accuracy of estimated breeding values (EBV) compared to parental average EBV, and increases response to selection for survival time compared to a traditional breeding scheme. We tested this hypothesis in two lines of brown layers with intact beaks, which show cannibalism, and also the hypothesis that the rate of inbreeding per year is lower for genomic selection than for the traditional breeding scheme.

**Results and discussion:**

The standard deviation of genomic prediction EBV for survival time was around 22 days for both lines, indicating good prospects for selection against mortality in laying hens with intact beaks. Genomic prediction increased the accuracy of the EBV by 35 and 32 % compared to the parent average EBV for the two lines. At the current reference population size, predicted response to selection was 91 % higher when using genomic selection than with the traditional breeding scheme, as a result of a shorter generation interval in males and greater accuracy of selection in females. The predicted rate of inbreeding per generation with truncation selection was substantially lower for genomic selection than for the traditional breeding scheme for both lines.

**Conclusions:**

Genomic selection for socially affected traits is a promising tool for the improvement of survival time in laying hens with intact beaks.

**Electronic supplementary material:**

The online version of this article (doi:10.1186/s12711-016-0247-4) contains supplementary material, which is available to authorized users.

## Background

Mortality due to cannibalism is an economic and welfare problem in laying hens, which reduces survival time [1]. Beak trimming and genetic selection are two strategies to reduce cannibalism and increase survival time. Although genetic selection to increase survival time has been implemented, responses to selection have been limited, in part because the heritability of the trait is low (around 0.02 to 0.10) [2]. Moreover, survival of laying hens that show cannibalism depends on social interactions among cage mates and may have a heritable component [3–6]; such social interactions are modelled as the so-called indirect genetic effects (IGE; [7]). Ignoring heritable components due to social interactions decreases response to selection, and may even cause a negative response to selection [3].

Recently, genetic selection methods for socially affected traits have become more efficient by taking IGE among cage mates into account [2, 4, 8, 9]. In laying hens that show cannibalism, accounting for IGE increases the detected heritable variation two to five times compared to the classical direct additive genetic variance [2, 10]. Although survival time has considerable heritable variation when IGE are accounted for, genetic improvement of survival time in laying hens is still challenging. First, heritable variation of the trait is relatively low, even with IGE (the proportion of total heritable variation to phenotypic variation ranges from 0.06 to 0.26). Second, and more importantly, survival time records are available late in life and only for females and many individuals are censored, i.e., still alive at the end of the testing period [2, 11]. Third, breeding females are kept in single bird cages and, therefore, own performance records for survival time under field conditions, i.e., measured in cages with multiple birds, are not available on female selection candidates. Thus, selection of females for survival time is based on pedigree and sib information, which leads to limited accuracy of selection. Selection of males for survival time also relies on information on the relatives, mainly from progeny information, which leads to a long generation interval. Consequently, response to selection for survival time is expected to be low. Thus, we need a better genetic tool, such as genomic selection, to increase response to selection.

Genomic selection is a genetic selection method in which genotypes at single nucleotide polymorphisms (SNPs) that cover the whole genome are used, so that all quantitative trait loci are expected to be in linkage disequilibrium with at least one SNP [12], and this information is used to predict breeding values. Genomic selection can increase the response to selection compared with traditional selection because genomic selection can increase the accuracy of estimated breeding values (EBV), particularly when compared with the parent average EBV [13–16], and genomic selection can reduce generation intervals compared to, e.g., schemes based on progeny testing. Thus, genomic selection schemes can result in greater response to selection per year compared with traditional breeding programs. For instance, in dairy cattle, genomic selection was predicted to increase genetic gain by 50 to 100 % [17, 18], and realized genetic progress in milk yield has been estimated to have increased by approximately 50 % in US Holsteins [19].

Currently, breeding programmes for laying hens are changing from progeny testing to genomic selection, particularly because it allows a substantial reduction in generation interval. Response to selection per year for egg number using genomic selection is expected to be higher than with progeny testing [20, 21]. A relevant question is whether genomic selection will also work for survival time in laying hens showing cannibalism, compared with the traditional breeding scheme, which refers to a scheme where males are selected based on progeny testing and females are selected based on sib and pedigree information.

Genomic evaluation can be implemented using genomic best linear unbiased prediction (GBLUP), for which a relationship matrix based on markers is used in BLUP [22, 23]. In this study, we estimated genomic breeding values using information on individuals that are both genotyped and phenotyped, as well as individuals that are phenotyped only. To exploit information on individuals that are phenotyped only in genomic evaluation, single-step GBLUP (ssGBLUP) was developed [24]. This procedure combines the relationship matrix derived from pedigree (**A**) and from genome-wide markers (**G**) into a single relationship matrix (**H**) [24–26]. The accuracy of EBV with correct blending of **G** and **A** for ssGBLUP is higher than with either GBLUP or pedigree-based BLUP [27].

Genetic selection with IGE coupled with genomic information may increase the response to selection in survival time for layers, compared with selection on pedigree-BLUP EBV. This can be tested by comparing the accuracy of EBV for survival time from ssGBLUP versus pedigree-BLUP, as well as by comparing responses to selection and rates of inbreeding when using genomic selection versus a traditional breeding scheme. Thus, the objective of this study was to investigate whether genomic prediction increases the accuracy of EBV and response to selection for survival time compared to a traditional breeding scheme, using data on crossbred brown layers. We also investigated the impact of genomic selection on the rate of inbreeding in a layer breeding program.

## Methods

### Population and pedigree

Data were provided by the Institut de Sélection Animale B.V., the layer breeding division of Hendrix Genetics. Phenotypes were available on crossbred individuals. In total, there were nine crosses from two sire lines and nine dam lines (Table 1); 19,975 crossbred laying hens had B1 as sire line, and 10,910 had BD as sire line. Sires were mated to approximately eight dams and each dam produced ~five male and ~five female offspring. Of each crossbred individual, only the sire ID was recorded; dam ID were unknown. Analyses were performed by sire line.

**Table 1:** Numbers of individuals and sires in the different crossbred populations analyzed

Cross (♂ × ♀)	Number of individuals	Number of sires	Number of genotyped sires
B1 × BA	3570	93	68
B1 × BB	1270	34	30
B1 × BD	5735	149	20
B1 × BE	1365	35	31
B1 × BF	4715	121	58
B1 × BH	3100	77	0
BD × B1	790	18	3
BD × B5	5415	150	138
BD × B6	4705	116	101

Data were collected in eight batches from 2008 to 2011 (Tables 2, 3). Post-hatching, the chicks were wing-banded, sexed, and vaccinated for infectious bronchitis and Marek’s disease. Their beaks were kept intact. At approximately 17 weeks of age, hens were placed in laying houses with battery cages. Battery cages were arranged in rows, each row with multiple levels. Cages consisted of five paternal half-sibs from the same cross. We removed cages with less than five individuals at the start of the experiment from the data (81 cages for line BD and no cages for line B1).

**Table 2:** Number of individuals censored (Nb ind) at different censoring points for line BD by batch

Batch	Nb ind	Nb ind ≥351 days	Nb ind ≥372 days	Nb ind ≥413 days
201042	5122	2461	0^b^	0
201182	4525	2694	2774	0
2009191	6385	3772	3673	3540
Total	16,032^a^	8927	6447	3540

^a^Total number of individuals without removing any batch. The ≥sign refers to individuals that were still alive at this censoring point. For example, for batch 201042, 2461 individuals were still alive at 351 days

^b^With a censoring point of 372 days, batch 201042 was removed. Thus, the number of individuals that remained for analysis was 4525 + 6385 = 10,910

**Table 3:** Number of individuals (Nb ind) censored at different censoring points for line B1 by batch

Batch	Nb ind	Nb ind ≥372	Nb ind ≥395	Nb ind ≥414	Nb ind ≥419	Nb ind ≥421
2008102	5397	3241	3129	3055	3027	0
200961	5228	3783	3711	3644	3617	3617
201124	5692	4181	4070	3933	0	0
201182	4981	3503	0^b^	0	0	0
2012102	3658	2675	2616	0	0	0
Total	24,956^a^	17,383	13,526	10,632	6644	3617

^a^Total number of individuals without removing any batch. The ≥ sign refers to the individuals that were still alive at this censoring moment. For example, for batch 2008102, 3241 individuals were still alive at 372 days

^b^With a censoring point of 395 days, batch 201182 was removed. Thus, the number of individuals that remained for analysis was 5397 + 5228 + 5692 + 3658 = 19,975

The trait of interest, “survival time”, was defined as “the number of days from the start of the laying period till either death or the end of the experiment”. The different batches had different censoring points (Table 2 for BD and Table 3 for B1). To avoid an effect of censoring at different time points on the analysis, we decided to censor all batches at the same time. Note that, with a proper survival analysis, the choice of a censoring point would have been irrelevant. However, Ellen et al. [28] found no benefit of survival analysis compared to a linear model for the analysis of survival time in laying hens when censoring occurred at the same time point. Hence, rather than using survival analysis, we decided to censor all batches at the same time point. Setting a common censoring point for all batches results in a trade-off between the proportions of censored individuals within the data and the amount of data included. On the one hand, choosing an early censoring point leads to many censored individuals. On the other hand, when choosing a very late censoring point, batches that have ended before this time point cannot be included in the analysis. We did not choose the common censoring points based on either the shortest batch, or the longest batch, which both would have resulted in the censoring or removal of many individuals. Therefore, for line BD, we took 372 days as the censoring point, so that 40 % of the individuals were censored and 32 % of the data were lost. For line B1, we took 395 days as the censoring point, which resulted in 54 % of the individuals being censored and 20 % of the data being lost (Table 2).

### Genomic data

Genotypes were available for part of the sires, i.e., 207 of the 509 B1 sires and 242 of the 284 BD sires were genotyped, both with 60 k SNP chips. The following quality controls were performed, separately for each line. Markers with a call rate less than 90 % or with a minor allele frequency (MAF) of 2 % or less were excluded. Setting the MAF at 2 % gave the highest accuracy of EBV compared to excluding SNPs with MAF <1, 3, 4, or 5 %. SNPs with a χ^2^ statistic greater than 600 for deviation from Hardy–Weinberg equilibrium were excluded (P value <0.00001). After quality control, a total of 35,361 SNPs for line B1 and 33,898 SNPs for BD remained.

### Data analysis

Data for each sire line were analysed separately. To determine which fixed effects should be included in the model to estimate genetic parameters, data on survival time were analysed using the GLM procedure in R [29]. The fixed effects of batch, cross and a contemporary group effect of laying house by row by level were fitted. The latter also accounts for infrastructural effects (e.g., differences in light intensity).

Because dam ID were unknown, sire models were fitted. The sire model only included a “direct” sire effect but, because cages consisted of paternal half sibs, this effect captures the total sire effect, including indirect genetic effects (IGE) [30]. The model was as follows:

1 $${\mathbf{y}} = {\mathbf{Xb}} + {\mathbf{Wc}} + {\mathbf{Zu}} + {\mathbf{e}},$$

where **y** is a vector of survival times, **X** is the incidence matrix for fixed effects, **b** is the vector of the aforementioned fixed effects, **c** is a vector of random group effects (i.e., cage effects), with ${\mathbf{c}} \sim N\left( {0,{\mathbf{I}}_{c} {\upsigma }_{c}^{2} } \right),\;{\mathbf{I}}_{c}$ is an identity matrix, **W** an incidence matrix for cage, ${\upsigma }_{c}^{2}$ is the cage variance, **Z** is an incidence matrix for the additive sire genetic effect, **u** is vector of sire-effects, and **e** is a vector of residuals. Two methods were used to implement this model.

*Method 1* was pedigree-BLUP, where the breeding values were assumed normally distributed as: ${\mathbf{u}} \sim N\left( {0,{\mathbf{A}}{\upsigma }_{u}^{2} } \right),$ where **A** is the genetic relationship matrix derived using five generations of pedigree information, and ${\upsigma }_{u}^{2}$ is the sire variance, which is equal to one-fourth of the variance of the total breeding values.

*Method 2* was ssGBLUP, where breeding values were assumed normally distributed as ${\mathbf{u}} \sim N\left( {0,{\mathbf{H}}{\upsigma }_{u}^{2} } \right),$ where **H** is the relationship matrix that combines both pedigree and genomic relationships. The inverse of **H** was obtained as [26, 31]:

2 $${\mathbf{H}}^{ - 1} = {\mathbf{A}}^{ - 1} + \left[ {\begin{array}{*{20}l} 0 \hfill & 0 \hfill \\ 0 \hfill & {\left( {{\upalpha }{\mathbf{G}} + {\upbeta }{\mathbf{A}}_{22} } \right)^{ - 1} - {\mathbf{A}}_{22}^{ - 1} } \hfill \\ \end{array} } \right],$$

where ${\mathbf{A}}^{ - 1}$ is the inverse of the numerator relationship matrix, **G** is the genomic relationship matrix, which was constructed following Method 1 of VanRaden [23] using the observed allele frequencies, and ${\mathbf{A}}_{22}$ is the sub-matrix of **A** for genotyped animals. We avoided singularity problems by regressing **G** towards **A**, by weighting ${\mathbf{G}}\left( {{\upalpha } = 0.95} \right)$ and ${\mathbf{A}}_{22} \left( {{\upbeta } = 2 - {\upalpha } = 0.05} \right)$ [23].

Genetic parameters and breeding values were estimated by residual maximum likelihood using the programme BLUPF90 [32, 33]. We did not use the “sire-model” option in BLUPF90 because it is not compatible with marker information. Instead, the phenotypes of hens were linked to the sires’ breeding values but we used a complete relationship matrix, including the full pedigree of the sires (for Method 1) or the full pedigree and the genotypes of the sires (for Method 2).

### Cross-validation

We considered four scenarios for estimating breeding values for each sire line; two types of reference training populations (genotyped sires only or both genotyped and non-genotyped sires), and two estimation methods (Methods 1 and 2) and compared the accuracy of predicted breeding values using cross-validation. We produced five mutually exclusive validation datasets by randomly sampling approximately 20 % of the genotyped sires (n = 207 for B1 and n = 242 for BD) without replacement. For each validation dataset, the remaining 80 % of the dataset served as the training dataset, which was used to estimate the breeding values for all individuals. For the scenario with both genotyped and non-genotyped sires, all non-genotyped sires were added to each training set.

Cross-validation requires observed phenotypes on the individuals in the validation dataset but, in our case, EBV were predicted on the sires, while records were available on offspring, and part of the offspring had censored records. Therefore, the following steps were taken to estimate the accuracy of EBV based on the Spearman rank correlation between the true and estimated breeding value of the sires in the validation set following [28]. First, the observed phenotypes were adjusted for fixed effects, using a linear model that included only the fixed effects $\left( {{\mathbf{y}} = {\mathbf{Xb}} + {\mathbf{e}}} \right)$ and residuals from this model served as corrected phenotypes for non-censored records. Second, the corrected phenotypes of the non-censored individuals were ranked from low to high survival time and censored records were assigned random ranks greater than the highest rank of the censored records. Then, the ranks of the censored records were replaced by the mean rank of the censored records [28]. Third, the “observed” rank of the sire was calculated as the mean observed rank of its daughters. Fourth, the correlation between the ranks of the estimated breeding values of sires and the observed ranks of the sires was calculated. This procedure was repeated for each validation set. The standard error of the correlation for each validation set was computed using $SE\left( {\hat{r}} \right) = \frac{{1 - \hat{r}^{2} }}{sqrt\left( n \right)}$, where *n* refers to the number of individuals in the validation set and $\hat{r}^{2}$ to the estimated squared correlation between the predicted and observed ranks of sires [34]. The rank correlation between predicted and observed sire ranks across the five sets, which was denoted as $\rho_{{\hat{A}_{s,} \bar{P}_{off} }} ,$ and its standard error was estimated as the residual correlation from a bivariate analysis of predicted and observed sire ranks, with validation set as the only fixed effect, using the ASREML software [35]. Finally, based on path-coefficients, the correlation between the EBV of a sire based on the training data and the mean performance of its offspring in the validation data is the product of the accuracy of the sire EBV $\left( {\rho_{{A_{s} ,\hat{A}_{s} }} } \right)$ and the correlation of the true sire breeding value with the offspring mean $\left( {\rho_{{\hat{A}_{s} ,\bar{P}_{off} }} } \right)$: $\rho_{{\hat{A}_{s} ,\bar{P}_{off} }} = \rho_{{A_{s} ,\hat{A}_{s} }} \rho_{{A_{s} ,\bar{P}_{off} }}$ [28]. Thus the accuracy of EBV of sires was calculated as:

$$\rho_{{A_{s} ,\hat{A}_{s} }} = \frac{{\rho_{{\hat{A}_{s} ,\bar{P}_{off} }} }}{{\rho_{{A_{s} ,\bar{P}_{off} }} }}.$$

The accuracy of progeny testing, i.e., the correlation between the true total breeding value of the sire and its estimated total breeding value from data on a progeny group, was calculated using:

3 $$\rho_{{A_{s} ,\bar{P}_{off} }} = \sqrt {\frac{{\hat{\sigma }_{u}^{2} }}{{\hat{\sigma }_{{\bar{p}}}^{2} }}} ,$$

where $\hat{\sigma }_{{\bar{p}}}^{2}$ is the variance of the mean progeny phenotypes for survival time among sires and $\hat{\sigma }_{u}^{2}$ is the sire variance estimated from the linear model (see above). Equation 3 represents the square-root of the proportion of the variance in the progeny average that is explained by the sire [36].

### Response to selection

To investigate the benefit of genomic selection with the current reference population size, response to selection and rate of inbreeding were compared between a traditional breeding scheme and a genomic selection scheme. Response to selection was predicted using deterministic simulation based on selection index theory, using the SelAction software [37]. SelAction predicts response and accuracy of selection for breeding programmes while accounting for the reduction in variance due to selection, known as the “Bulmer effect” [38]. This is essential when comparing genomic selection and traditional breeding programs, particularly when accuracies differ substantially between the sexes, which is the case in traditional breeding schemes in laying hens [16, 39, 40]. Note that, since selection is for a single trait here (survival time), accuracy of EBV and accuracy of selection are the same thing. However, we interpreted the EBV accuracies from cross-validation as referring to an unselected population, whereas accuracies from SelAction are reduced by the Bulmer-effect. Hence, results given below refer to response and accuracy for a population after reaching Bulmer equilibrium [38].

For the breeding schemes, the inputs shown in Table 4 were used in SelAction (provided by Hendrix genetics). We used selected proportions of 8 % in males and females for the traditional breeding scheme, and of 2 % in males and 8 % in females for the genomic selection scheme for both lines. We used 20 breeding males and 400 breeding females per generation for both the genomic selection and the traditional breeding scheme for both lines. Each sire was mated to 20 dams and each dam produced five male and five female offspring. The generation interval for the traditional breeding scheme was 99 weeks for males and 55 weeks for females for both lines. The generation interval for the genomic selection scheme was 33 weeks for males and 55 weeks for females for both lines. In the traditional breeding scheme, males were selected based on pedigree information and on the average phenotype of 40 progeny born from eight dams, while females were selected on pedigree information only for both lines, because female selection candidates are kept in individual cages and therefore do not have own performance records on survival in group housing. Also, sib information on survival time is not available for females at the time of selection. In the genomic selection scheme, both male and female selection candidates were genotyped and selected based on their GEBV.

**Table 4:** Inputs used to estimate response to selection and rate of inbreeding using SelAction

Input	Progeny testing	Genomic selection
Selected proportion for males	8 %	2 %
Selected proportion for females	8 %	8 %
Generation interval for males	99 weeks	33 weeks
Generation interval for females	55 weeks	55 weeks
Information used for males	Parental average, progeny (40)	Own^a^
Information used for females	Parental average	Own^a^
Number of sires (dams)	20 (400)	20 (400)

Genetic parameters required for SelAction were taken from the results presented in Table 5 (see below), averaged over both lines

^a^Own indicates that the selection candidates are genotyped

For the genetic parameters of survival time, inputs were the averages of the estimates for lines B1 and BD, taken from Table 5 (the input values used are given in the footnote of Table 7). These inputs are sufficient for the traditional breeding scheme, which was modelled as single-trait selection. The genomic selection scheme was simulated by adding a correlated trait with full heritability, representing the marker information [41, 42]. Only survival time was included in the breeding goal; the economic value of the marker information was zero. SelAction input and output files are included as additional information (see Additional file 1, Additional file 2, Additional file 3, Additional file 4).

**Table 5:** Estimated variance components for survival time for lines B1 and BD, using pedigree relationships

Variance component	Line B1	Line BD
${{\hat{\varvec{\upsigma }}}}_{{\mathbf{e}}}^{{\bf 2}}$	8885 ± 99	10,350 ± 156
$\hat{\varvec{\sigma }}_{c}^{{\bf 2}}$	1084 ± 72	1403 ± 118
${{\hat{\varvec{\upsigma }}}}_{{{\mathbf{A}}_{{\mathbf{T}}} }}^{{\bf 2}}$	1912 ± 244	2700 ± 424
$\hat{\varvec{\sigma }}_{P}^{{\bf 2}}$	10,446 ± 115	12,428 ± 189
${{\hat{\varvec{\upsigma}}}}_{{{\bar{\mathbf{P}}}_{{{\mathbf{off}}}} }}^{{\bf 2}}$	1327 ± 56	1280 ± 80
${\hat{\mathbf{T}}}^{{\bf 2}}$	0.18 ± 0.02	0.22 ± 0.03

${{\hat{\varvec{\upsigma}}}}_{{{\mathbf{A}}_{{\mathbf{T}}} }}^{{\bf 2}} = 4{{\hat{\varvec{\upsigma}}}}_{{\mathbf{u}}}^{{\bf 2}}$ is the total additive genetic variance, including both direct and the indirect components [43]

${{\hat{\varvec{\upsigma} }}}_{{{\bar{\mathbf{P}}}_{{{\mathbf{off}}}} }}^{{\bf 2}}$ is the variance of the mean progeny phenotype among sires. Its standard error is computed as ${{\hat{\varvec{\upsigma} }}}_{{{\bar{\mathbf{P}}}_{{{\mathbf{off}}}} }}^{{\bf 2}} \sqrt {\frac{2}{n - 1}}$, *n* denoting the number of sires

${\hat{\mathbf{T}}}^{{\bf 2}} = {{\hat{\varvec{\upsigma}}}}_{{{\mathbf{A}}_{{\mathbf{T}}} }}^{{\bf 2}} /{\hat{\varvec{\sigma }}}_{P}^{{\bf 2}}$ represents total additive genetic variance as a proportion of the phenotypic variance

$\hat{\varvec{\sigma }}_{P}^{{\bf 2}} = 4{{\hat{\varvec{\upsigma}}}}_{{\mathbf{u}}}^{{\bf 2}} + \hat{\varvec{\sigma }}_{c}^{{\bf 2}} + {{\hat{\varvec{\upsigma}}}}_{{\mathbf{e}}}^{{\bf 2}}$ where $\hat{\varvec{\sigma }}_{c}^{{\bf 2}}$ is the cage variance and ${{\hat{\varvec{\upsigma}}}}_{{\mathbf{e}}}^{{\bf 2}}$ is the residual variance

For the genomic selection scheme, the key input parameter for SelAction is the genetic correlation between the marker information and survival time, $r_{g} ,$ because this parameter determines the accuracy of the GEBV for survival time in the genomic selection scheme. The input value for $r_{g}$ was based on the relative accuracies of genomic versus traditional EBV observed in the cross-validation. Thus, SelAction was used twice. First, to find the $r_{g}$ that agrees with the cross-validation, and second to predict response to selection for the genomic selection scheme. In the second run, the $r_{g}$ of the first run was used as input. The following steps were taken to find the value of $r_{g}$ that agreed with the results of the cross-validation:

1. The accuracy of the parent-average EBV, $\rho_{PA} ,$ for the traditional breeding scheme was obtained with SelAction, considering a population without selection (selected proportions of 100 % in both sexes). Thus, a single trait analysis was done in SelAction, including only survival time. This yielded $\rho_{PA} = 0.45.$
2. The target accuracy of the genomic selection scheme, $\rho_{GS} ,$ was obtained by multiplying $\rho_{PA}$ from step 1 with the ratio of the accuracy of GS over that of the traditional scheme, as found in the cross-validation (Table 6). This ratio was ~1.33, so the target accuracy of GS was $\rho_{GS} = 0.45 \times 1.33 = 0.60.$

**Table 6:** Cross-validation results for lines B1 and BD, with genotyped sires as reference population for ssGBLUP and pedigree-BLUP

Cross-validation fold^a^	SsGBLUP	Pedigree-BLUP	SsGBLUP	Pedigree-BLUP
	Line B1		Line BD	
1st 20 %	0.16 ± 0.15	0.08 ± 0.15	0.36 ± 012	0.23 ± 0.14
2nd 20 %	0.38 ± 0.12	0.17 ± 0.14	0.30 ± 0.13	0.19 ± 0.14
3rd 20 %	0.30 ± 0.15	0.21 ± 0.16	0.29 ± 0.13	0.11 ± 0.14
4th 20 %	0.44 ± 0.12	0.43 ± 0.13	0.16 ± 0.14	0.18 ± 0.14
5th 20 %	0.43 ± 0.13	0.42 ± 013	0.25 ± 0.14	0.27 ± 0.13
Average	0.35 ± 0.06	0.26 ± 0.07	0.27 ± 0.06	0.20 ± 0.07
Accuracy^2^	0.58 ± 0.08	0.43 ± 0.09	0.37 ± 0.08	0.28 ± 0.09

Values are the correlations of the estimated breeding values of sires with the average phenotype of their offspring, ($\rho_{{\hat{A},\bar{P}_{off} }}$)

^a^Accuracy of the estimate true breeding value ($\rho_{{A_{s} ,\hat{A}_{s} }}$); see Eq. 3
3. The required genetic correlation between the marker information and survival time, $r_{g}$, was found iteratively in SelAction by varying $r_{g}$ until the accuracy of selection reached 0.60. Thus, a two-trait analysis was done in SelAction, including both survival time and the marker information. This yielded $r_{g} = 0.53.$

The next step was to predict response to genomic selection using $r_{g} = 0.53$ as input for SelAction. We aimed at predicting response for the case when selection candidates have no close relatives in the reference population for two reasons: (1) to be conservative, and (2) although reference populations will be updated over time, there is no guarantee that a substantial proportion of the reference individuals will be closely-related to the selection candidates. However, in the data used for cross-validation (see above), the majority of the validation sires had fathers with female progeny with records on survival time. Thus, the validation sires had half-sibs with phenotypic information in the reference population. Hence, in ssGBLUP, the cross-validation accuracy resulted not only from genomic information but also from close pedigree relationships with individuals in the reference population. To extract the contribution of marker information to the accuracy of cross-validation, we used progeny-tested sires in step 3. Hence, the accuracy of 0.60 in step 3 refers to individuals that had both progeny-tested fathers and marker information, in a population without selection. This resembles the situation in cross-validation and explains why the required $r_{g}$ of 0.53 was smaller than 0.60, because progeny testing of the fathers also contributed to the 0.60 accuracy. Simply using $r_{g} = \rho_{GS} = 0.60$ would over-predict response to GS, as it would attribute the full cross-validation accuracy to the marker information. In the final SelAction run that was used to predict response for the GS-scheme, there was no progeny testing of males, because we assumed that selection candidates had no close relatives in the reference population. Hence, the initial (i.e., unselected) accuracy for that scheme was equal to $r_{g} = 0.53,$ and thus lower than the value of 0.60 found in cross-validation. In the Results, we present responses for the Bulmer-equilibrium situation.

## Results

**Fig. 1:**
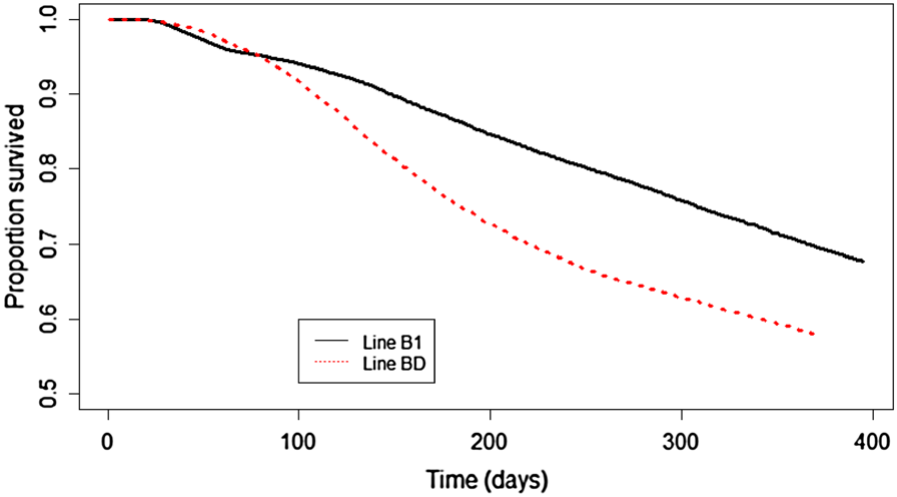
Proportion of surviving individuals

Both lines showed considerable mortality (Fig. 1) but the average survival time for line B1 was higher than for line BD. A significant effect on survival time was found for cross, batch, and laying house*row*level for both lines (P < 0.001).

Table 5 shows the variance components estimated using statistical Method 1 (pedigree BLUP). The total heritable variation relative to phenotypic variance, ${\text{T}}^{2} ,$ was 0.18 for line B1 and 0.22 for line BD. The estimated total genetic standard deviation was ~44 days for line B1 and ~52 days for line BD, which were significantly different from zero (P < 0.001) and indicate good prospects for genetic improvement. Note that this estimate includes both direct and indirect genetic effects.

Table 6 shows the correlations of the EBV of sires with the mean corrected rank of their offspring, calculated from cross-validation, when only the genotyped sires were used. The correlation of the EBV of sires with the mean phenotype of their offspring was higher for ssGBLUP than for pedigree-BLUP, for both lines. With ssGBLUP, accuracies of EBV were 35 and 33 % higher for lines B1 and BD, respectively, compared to parental average (0.58 vs. 0.43 for line B1, and 0.37 vs. 0.28 for line BD). Correlations obtained with ssGBLUP were only slightly higher when non-genotyped sires were also included in the reference population [0.62 vs. 0.45 for line B1, and 0.39 vs. 0.28 for line BD (see Additional file 5: Table S1)].

Table 7 shows predicted responses to selection per year and predicted rates of inbreeding per generation and per year. Predicted response for the genomic scheme was 91 % higher than that of the traditional scheme, primarily because of a reduction in the generation interval of males and a greater accuracy of selection for females. The predicted rate of inbreeding per year was 53 % lower for the genomic scheme than for the traditional scheme.

**Table 7:** Predicted accuracy and response to selection in survival time and rate of inbreeding

	Traditional	Genomic selection
Accuracy of males	0.77	0.42
Accuracy of females	0.16	0.42
Response to selection (days/year)	24.6	47.0
Rate of inbreeding (%) per generation (year)	2.75 (1.86)	0.75 (0.88)

Values refer to the Bulmer equilibrium; inputs are in Table 4

Additional inputs (unselected base population parameters averaged over lines are from Table 5): $\hat{\varvec{\sigma }}_{\varvec{P}}^{2} =$ 11,500 days^2^ and $\varvec{h}^{2} =$ 0.20

For the GS scheme: genetic correlation between survival time and marker information $\varvec{r}_{\varvec{g}} = 0.53$

Corresponding phenotypic correlation $\varvec{r}_{\varvec{p}} = \varvec{hr}_{\varvec{g}} = 0.24$

Heritability marker information = 100 %, and phenotypic variance marker information = 646.7 days^2^; the latter is obtained as $\varvec{r}_{\varvec{g}}^{2} \varvec{h}^{2}\varvec{\sigma}_{\varvec{P}}^{2} ,$ which causes the regression coefficient of the true breeding value for survival time on the marker-based EBV to be equal to 1. Note that this value does not impact response to selection in survival time. Further details are in [41, 42]

## Discussion

We show that genomic selection increases the accuracy of EBV for survival time in brown layers compared to the parent average EBV (Table 6). More importantly, although the reference population is currently small, genomic selection resulted in a substantially higher response to selection per year for survival time compared to the traditional breeding scheme. The standard deviation in genomic EBV was 25 days for line B1 and 19 days for line BD, indicating good prospects for selection against mortality due to cannibalism in these brown layer lines.

### Genetic parameters

The structure of the design with cages consisting of paternal half-sibs makes it possible to directly estimate the linear combination of the direct and indirect breeding values, which is the total breeding value [30, 43], but the separate contributions of direct and indirect genetic effects to the total genetic variance cannot be estimated [30]. Using pedigree relationships, the proportion of the total heritable variation to phenotypic variance for survival time was 0.18 for line B1 and 0.22 for line BD. Similar total heritabilities for survival time (0.1–0.2) were found in purebred white layers by Ellen et al. [2] and in crossbred white layers by Peeters et al. [11]. In white layer lines, the indirect genetic variance contributes the majority of the total genetic variance [2, 11]. The total heritability for survival time was considerably higher than common heritabilities (0.02–0.1) in white layers [2, 11]. If this result extends to brown layers, then indirect genetic effects also contribute substantially to the heritable variance in survival time in our populations.

### Accuracy of EBV

We found that the accuracy of genomic EBV was ~33 % higher than that of parent average EBV in both lines. The absolute increases in accuracy were 0.15 for line B1 and 0.09 for line BD. While the relative superiority of genomic selection over the traditional scheme was similar for both lines, the absolute accuracies were clearly higher for line B1 than for line BD, both for pedigree BLUP and for ssGBLUP. In the following, we discuss the mechanisms that may cause these differences in accuracy. First, we consider the contribution of pedigree information, then the contribution of genomic information, and finally the joint contribution of both information sources to the accuracy of EBV.

The large difference in the accuracy of parent average EBV between lines suggests that line B1 benefits more from pedigree information than line BD. The key factor that determines the accuracy of EBV based on information on relatives is the variation in relatedness among pairs of individuals rather than the average level of relatedness, see [44]. The variance in pedigree relationships, i.e., the variance of the off-diagonal elements of the pedigree-relationship matrix among the sires of each line (509 for B1 and 284 for BD) was 0.00091 for line B1 and 0.00077 for line BD. Thus, the variation in pedigree relationships was larger for line B1 than for line BD, which agrees with the higher accuracies of the parent average EBV for line B1.

With genomic information, the accuracy of EBV obtained with GBLUP depends on the average linkage disequilibrium (LD) across the genome, the number of observations in the reference population, and the reliability of the information source recorded in the reference population [44–47]. Without LD, all loci would segregate independently and genomic relationships would be identical to pedigree relationships [44]. Hence, variation of genomic relationships around their expectation based on pedigree reflects LD, with LD being greater if this variation is greater, resulting in greater accuracy of GEVB compared to EBV based on pedigree information. Thus, the genome-wide average LD can be measured by ${\text{var}}\left( {{\mathbf{G}} - {\mathbf{A}}_{22} } \right),$ which denotes the variance of the difference between the pedigree relationship and the genomic relationship, taken over all pairs of individuals (genotyped sires here [44]). The reciprocal of the variation in genomic relatedness, $M_{e} = \frac{1}{{{\text{var}}\left( {{\mathbf{G}} - {\mathbf{A}}_{22} } \right)}},$ is a measure of the effective number of independently segregating chromosome segments, and represents the effective number of independent genetic effects that have to be estimated in genomic prediction [44, 46]. As $M_{e}$ increases, the genome-wide average LD decreases, resulting in a lower accuracy of GEBV [44, 46, 48].

To quantify genome-wide average LD in each line, we calculated $M_{e}$ based on the genotyped sires of each line, resulting in $M_{e} = 799$ for B1 and $M_{e} = 1020$ for BD. Thus, variation in genomic relationships around their expectation based on pedigree was greater for line B1 than for line BD, which reflects that the LD was greater for line B1 than for line BD [44].

With genomic information only, i.e., in the absence of close pedigree relationships, the theoretically expected accuracy of GEBV equals (Eq. 1 in [45, 46]; Appendix A in [47]):

4 $$r_{{\hat{A}A}} = \sqrt {\frac{{N_{p} r^{2} }}{{N_{p} r^{2} + M_{e} }}} ,$$

where $r^{2}$ refers to the reliability of a phenotypic observation in the reference population, and $N_{p}$ to performance of animals in the reference population. Because our reference population consisted of progeny-tested sires, we used the reliability of progeny testing, which equals the square of the accuracy of progeny testing given in Eq. 3. Reliabilities of progeny testing, calculated from values in Table 5, were $r_{B1}^{2} = 0.36$ and $r_{BD}^{2} = 0.53.$ Substituting those values in Eq. 4 yielded theoretically expected accuracies of 0.26 for line B1 and 0.30 for line BD. Thus, the contribution of genomic information to the accuracy of EBV was similar for both lines; it was slightly higher for line BD than for line B1, because the larger reference population and the higher value of $r^{2}$ for BD more than compensate for the lower LD in that line.

Finally, we quantified the contributions of pedigree and genomic information to the empirical accuracy of EBV from ssGBLUP observed in cross-validation ($\rho_{{A\hat{A}}}$, Table 6). For line B1, the theoretically expected accuracy was 0.26 and the empirical accuracy was 0.58. For line BD, the theoretically expected accuracy was 0.30 and the empirical accuracy was 0.37. Hence, empirical accuracies were higher than theoretically expected accuracies, particularly for line B1. This demonstrates that pedigree relationships also contributed to the accuracy of GEBV. The greater contribution of close pedigree relationships for line B1 agrees with the greater variation in pedigree relationships and the greater accuracy of parent average EBV for line B1.

### Rate of inbreeding

The predicted rate of inbreeding of ~1.86 % per year for the traditional breeding scheme was considerably larger than the 0.88 % per year for the genomic scheme (Table 7). Note that these rates of inbreeding refer to a breeding scheme where parents are selected solely by truncation based on their total longevity EBV, without any restriction on the number of individuals selected from the same family or on relatedness between selected individuals. Rates of inbreeding with truncation selection were predicted using the theory of long-term genetic contributions [49], as implemented in SelAction [37].

The difference in rates of inbreeding between the traditional and genomic breeding schemes is due to two reasons. First, with the traditional breeding scheme, females are selected by truncation on parent average EBV, resulting in selection of complete families, which is known to increase the rate of inbreeding [50]. Second, with GS there is a strong Bulmer effect, since the GEBV has a heritability of 1, which reduces the rate of inbreeding [51]. The Bulmer effect reduces the between-family variance in EBV, which reduces the correlation between EBV of sibs and of more distance relatives. A lower correlation between EBV of relatives reduces the probability of co-selection of relatives, and causes selection to act more strongly within families, which reduces the rate of inbreeding. Together, these two mechanisms cause a considerable difference in the expected rates of inbreeding between the two schemes.

The predicted rate of inbreeding for the traditional scheme (~1.86 %/year) would be viewed as unacceptable in practice and, thus, measures would be taken to restrict it. Thus, parents would no longer be selected solely by truncation on EBV, which would cause a decrease in response to selection for the traditional scheme. Hence, with restricted inbreeding, compared to the GS scheme the traditional breeding scheme would yield even less response than suggested by the values in Table 7.

## Conclusions

Overall, in spite of the small reference population sizes (207 genotyped sires for line B1 and 242 for line BD), genomic selection showed a reasonably good accuracy for predicted true breeding values compared to pedigree-BLUP for survival time in brown layers. More importantly, it gave a substantially higher expected response to selection and lower rate of inbreeding compared to the traditional breeding scheme. Thus, for genetic improvement of survival time in laying hens that show cannibalism, genomic selection is an attractive alternative for traditional selection, even if the available reference population is small.

## Additional files

10.1186/s12711-016-0247-4 Input used for selAction software to predict response to selection and rate of inbreeding for traditional selection.
10.1186/s12711-016-0247-4 Input used for selAction software to predict response to selection and rate of inbreeding for genomic selection.
10.1186/s12711-016-0247-4 Output which includes predicted response to selection and rate of inbreeding from selAction software for traditional selection.
10.1186/s12711-016-0247-4 Output which includes predicted response to selection and rate of inbreeding from selAction software for genomic selection.
10.1186/s12711-016-0247-4 Cross-validation results for lines B1 and BD, with both genotyped and non-genotyped sires as reference population for ssGBLUP and pedigree-BLUP.
