# Supplementary material for: Genomic prediction of survival time in a population of brown laying hens showing cannibalistic behavior
Source: Genet Sel Evol. 2016 Sep 13;48(1):68. doi: 10.1186/s12711-016-0247-4 (PMC5022246; doi:10.1186/s12711-016-0247-4)
Supplement: Supplementary file 3 — 10.1186/s12711-016-0247-4 Output which includes predicted response to selection and rate of inbreeding from selAction software for traditional selection. [file 12711_2016_247_MOESM3_ESM.rtf]

 SelAction Version 2.1, licensed to Test licence, Marc Rutten and Piter Bijma
 These results were generated 22-4-2016, 13:11:51
 Using input from  File: M:\WRK\Aio's\Setegn\paper 4 genomic prediction brown lines\3rd submission\SelAction 
files\Traditional_prediction_response.d1s
 
  TRAITS USED
 
   survival_time
 
 
  TRAIT PARAMETERS
  
                 phenotypic variance  heritability
   survival_time    11,500.0000         0.2000
 
 
  BREEDING GOAL INFORMATION
 
         1.0000 * survival_time
 
 
  POPULATION SIZE
  
               number of selected male parents : 20
             number of selected female parents : 400
   number of male selection candidates per dam : 5.0
 number of female selection candidates per dam : 5.0
 
        total selected proportion male parents : 0.080
      total selected proportion female parents : 0.080
 
 CHARACTERISTICS OF THE USED GROUPS
 
  progeny group 1 with        8.0 dams, producing       40.0 progeny
 
 
 INDEX INFORMATION FOR MALE CANDIDATES :
 
           Dam BLUP breeding value on survival_time  
          Sire BLUP breeding value on survival_time  
   Observations on progeny group 1 on survival_time  
 
 INDEX INFORMATION FOR FEMALE CANDIDATES :
 
           Dam BLUP breeding value on survival_time  
          Sire BLUP breeding value on survival_time  
 
             ******************   RESULTS   *******************
 
 EQUILIBRIUM PARAMETERS
 
                 phenotypic variance  heritability
   survival_time    11,024.7410         0.1655
 
  RESPONSE
                                      males         females         total
   survival_time
               trait units:          30.507          5.855         36.362 (Beware, this is per generation)
            economic units:          30.507          5.855         36.362
        % of totalresponse:          83.898         16.102        100.000
 
  TOTALRESPONSE
                                      males         females         total
 
            economic units:          30.507          5.855         36.362 
 
 
         variance of index:       1,085.994         45.156
 variance of breeding goal:       1,824.746
         accuracy of index:          0.771          0.157
 
    rate of inbreeding:           2.750 % per generation
 
 
                      ******  end of output  ******
