# Supplementary material for: Genomic prediction of survival time in a population of brown laying hens showing cannibalistic behavior
Source: Genet Sel Evol. 2016 Sep 13;48(1):68. doi: 10.1186/s12711-016-0247-4 (PMC5022246; doi:10.1186/s12711-016-0247-4)
Supplement: Supplementary file 4 — 10.1186/s12711-016-0247-4 Output which includes predicted response to selection and rate of inbreeding from selAction software for genomic selection. [file 12711_2016_247_MOESM4_ESM.rtf]

 SelAction Version 2.1, licensed to Test licence, Marc Rutten and Piter Bijma
 These results were generated 22-4-2016, 13:33:56
 Using input from  File: M:\WRK\Aio's\Setegn\paper 4 genomic prediction brown lines\3rd submission\SelAction 
files\Genomic_prediction_response.d1s
 
  TRAITS USED
 
   survival_time
         genomic
 
 
  TRAIT PARAMETERS
  
                 phenotypic variance  heritability
   survival_time    11,500.0000         0.2000
         genomic       646.0700         0.9990
 
 
  PHENOTYPIC CORRELATIONS
  
       genom
 survi  0.24
 
  GENETIC CORRELATIONS
  
       genom
 survi  0.53
 
  BREEDING GOAL INFORMATION
 
         1.0000 * survival_time
 
 
  POPULATION SIZE
  
               number of selected male parents : 20
             number of selected female parents : 400
   number of male selection candidates per dam : 5.0
 number of female selection candidates per dam : 5.0
 
        total selected proportion male parents : 0.020
      total selected proportion female parents : 0.080
 
 
 
 INDEX INFORMATION FOR MALE CANDIDATES :
 
           Dam BLUP breeding value on survival_time  
          Sire BLUP breeding value on survival_time  
                   Own performance on genomic        
 
 INDEX INFORMATION FOR FEMALE CANDIDATES :
 
           Dam BLUP breeding value on survival_time  
          Sire BLUP breeding value on survival_time  
                   Own performance on genomic        
 
             ******************   RESULTS   *******************
 
 EQUILIBRIUM PARAMETERS
 
                 phenotypic variance  heritability
   survival_time    11,201.2069         0.1787
         genomic       347.7578         0.9981
 
  PHENOTYPIC CORRELATIONS
  
       genom
 survi  0.18
 
  GENETIC CORRELATIONS
  
       genom
 survi  0.42
 
  RESPONSE
                                      males         females         total
   survival_time
               trait units:          22.476         17.274         39.750 (Beware, this is per generation)
            economic units:          22.476         17.274         39.750
        % of totalresponse:          56.542         43.458        100.000
 
  CORRELATED RESPONSE
         genomic
               trait units:          22.464         17.266         39.730
 
  TOTALRESPONSE
                                      males         females         total
 
            economic units:          22.476         17.274         39.750
 
 
         variance of index:         346.814        346.814
 variance of breeding goal:       2,001.024
         accuracy of index:          0.416          0.416
 
    increase of inbreeding:           0.746 % per generation
 
 
                      ******  end of output  ******
