# Supplementary material for: Genomic prediction of survival time in a population of brown laying hens showing cannibalistic behavior
Source: Genet Sel Evol. 2016 Sep 13;48(1):68. doi: 10.1186/s12711-016-0247-4 (PMC5022246; doi:10.1186/s12711-016-0247-4)
Supplement: Supplementary file 5 — 10.1186/s12711-016-0247-4 Cross-validation results for lines B1 and BD, with both genotyped and non-genotyped sires as reference population for ssGBLUP and pedigree-BLUP. [file 12711_2016_247_MOESM5_ESM.docx]

|  | **Line B1** | | **Line BD** | |
| --- | --- | --- | --- | --- |
| **Method** | ssGBLUP | Pedigree-BLUP | ssGBLUP | Pedigree-BLUP |
| 1st 20% | 0.29±0.13 | 0.15±0.15 | 0.36±0.12 | 0.24±0.15 |
| 2nd 20% | 0.30±0.13 | 0.08±0.14 | 0.33±0.12 | 0.19±0.14 |
| 3rd 20% | 0.37±0.15 | 0.26±0.16 | 0.35±0.12 | 0.18±0.14 |
| 4th 20% | 0.48±0.12 | 0.42±0.13 | 0.14±0.12 | 0.18±0.14 |
| 5th 20% | 0.42±0.13 | 0.43±0.13 | 0.19±0.13 | 0.19±0.14 |
| **Average** | 0.37±0.06 | 0.27±0.07 | 0.28±0.06 | 0.20±0.06 |
| Accuracy^2^ | 0.62±0.08 | 0.45±0.09 | 0.39±0.08 | 0.28±0.08 |

^1^Values are the correlation of estimated breeding values of sires with the average phenotype of their offspring (), for ssGBLUP and pedigree-BLUP. ^2^The accuracy of estimating the true breeding value (); see above Equation 3.
